# HRM issues and practices in internal stakeholder management for healthcare: a bibliometric and systematic literature analysis

**DOI:** 10.1186/s12960-026-01049-z

**Published:** 2026-01-13

**Authors:** Lavender Awino Okore, Edina Molnár

**Affiliations:** 1 Faculty of Health Sciences, Doctoral School of Management and Business, University of Debrecen, Böszörményi út 138, Egyetem ter 1, 4032 Hungary; 2 Faculty of Health Sciences, Institute of Social Studies, University of Debrecen, Böszörményi út 138, Debrecen, 4032 Hungary

**Keywords:** Public health, Primary healthcare (PHC), Community health (CH), Health workers, Internal stakeholders

## Abstract

Effective human resource management (HRM) is a dominant topic of interest owing to its crucial role in both health production and service provision. Despite the recognition of the fundamental Strategic HRM (SHRM) practices, the intellectual structure and direction of research is yet to be fully developed, integrated and practically evolved in response to contemporary developments. Therefore, this study analyses the knowledge structure and evolution of HRM issues and practices in internal stakeholder management (ISM) within healthcare. In this study, we conduct a bibliometric analysis of 477 articles published in the Web of Science database from 2000 to 2024. The articles were synthesised through a qualitative analysis (citation network analysis and a concurrence network of key words). We synthesised 6 clusters that highlighted distinct themes and their respective references using VOS Viewer. Thereafter, a systematic analysis was conducted to identify gaps and synthesize findings. The findings revealed 6 major thematic clusters: (1) HRM Innovation and Work Engagement Practices; (2) Lean Management and Empowerment; (3) Cross-sectoral High Performance Work Systems (HPWS); (4) Work Environment and Patient Satisfaction Factors-high commitment work systems (HCWS); (5) Leadership and Employee Well-being; (6) HRM and Institutional Performance. The analysis confirmed employee well-being, leadership engagement, burn-out and the implementation of HPWS and HCWS as emerging issues in HRM practice. The findings evidence the evolution of HPWS towards a dual integrated model with employee well-being, centrally supported by leadership. We highlight the strategic framing significance of SDG 3 (Good Health and Well-being) and SDG 8 (Decent Work and Economic Growth), in the operationalisation and cascading of public and organisational HRM practices in healthcare. We argue out the managerial tension in managing efficiency and satisfaction based on the influential studies advancing performance and other dominant clusters revealing salience for human capital support. Therefore, the findings uphold the imperative of anchoring (SHRM) practices on engaging leadership, fair compensation practices, employee well-being, robust and responsive support systems. We contribute to enhanced ISM organisational performance and sustainable health-care delivery by bringing to the fore evidence-based insights that supports policy development and effective health-care management.

## Introduction

Rapid business environment developments resulting from emerging technologies, evolving social issues, and the ever-changing nature of work have become the order of the day [1]. The healthcare sector is not immune to these external changes, and the need for innovative approaches especially as new disease patterns, health production initiatives, growing diversity in patient demands, and recent on non-communicable diseases (NCDs) take the centre-stage of one of the most labour-intensive sectors globally [2]. Within such complexities, HRM can no longer be an operational and administrative function, as initially constructed, majorly in the public sector, instead, its global evolution towards strategic practices is inevitable. The perspectives in this review conceptualise the SHRM transition both as a predictable and unpredictable change management process that regardless is imperative for optimal healthcare service delivery. The interaction between SDG 3 and SDG 8 has been studied from a sustainable HRM perspective. Work has become a major public health risk due to the nature of working conditions and the elements used to manage unit labour costs for international competitiveness [3]. Over time, the health-related targets for SDG 3 have been adversely stretched by the demands of high-economic growth contexts posing even greater challenges for governments, multinational entities and practitioners trying to achieve sustainable health and well-being for employees as internal stakeholders [4].

Although there’s ongoing research in sectoral Human Resource Management, senior managers and executives are still struggling with infusing the soft aspects of work in their core implementation plans, over and above the technical preliminaries. To reach organisational strategic goals, conversations in academia and industry are underscoring the importance of people-centred approaches in the work environment, citing people’s efforts as the irreplaceable driving force, especially in patient care [5]. Concurrently, the efficacy of community health workers and health professionals has been widely covered in research. The health workforce (clinicians, nurses, doctors, volunteers) is an essential primary stakeholder in the health ecosystem [6, 7]. Contemporary issues in human resource management have unearthed employees’ pursuit to be centred around high-value engagements that place them better within the ecosystem, innovations, and industry pay nets [8, 9].

Theoretical advancements in SHRM underscore the critical role of engagement and balancing diverse interests and demands of employees as internal stakeholders. In understanding SHRM practices for ISM, we adopt the contingency perspective to dissect the architecture of HRM themes practices and relationship with outcomes in the healthcare sector. The SHRM perspective holds an HRM orientation that is driven by the organisations’ values, mission, the needs, goals and expectations of its stakeholders [10]. Additionally, the authors concluded that effective SHRM requires a holistic, sustained approach that is committed to involvement, leadership, diversity and deliberate effort to dismantle biases [11]. Despite theoretical and empirical grounding, there is paucity in literature across heath disciplines and fragmentation across different scopes of public health interventions (global, community, family) as well as sub-filed such as (policy, mental health and suicide prevention, health education). A bibliometric analysis fits because of its statistical capability to objectively map scientific literature patterns, reveal relevant thematic structure and evolution of research in a field. This level of synthesis, coupled with a descriptive scope of literature content, delivers structurally—fit mapping of the existing knowledge base. Therefore, the objective of the analysis was to conduct a Bibliometric and systematic literature review (SLR) of research on HRM Practices and managerial issues ISM establish how research on human resource practices and healthcare management are related and to corroborate the interconnectedness of the HR practice areas in health, based on existing literature.

- RQ1. What are the health human resourcing factors and dominant thematic research clusters that influence stakeholder management practices for performance in healthcare?
- RQ2. What main managerial approaches and issues are dominant in literature for effective health human resource stakeholder management?
- RQ3. Which SHRM practices that support optimal and sustainable healthcare services emerge from influential literature?

## Materials and methods

**Fig. 1:**
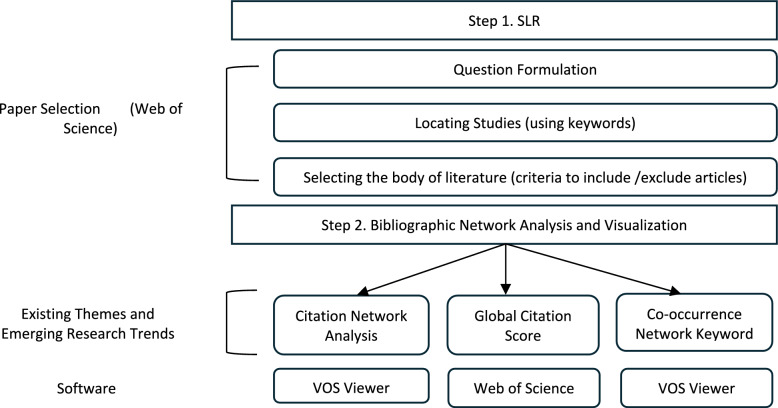
Study identification and selection process adopted from Máté et al. (12)

Bibliometrics and SLR were identified as some of the most effective tools for synthesising and addressing literature’s paucity, hence a high volume of quantitative data aimed at mapping the scientific intellectual structure in the field of HRM and health. Beyond the potential of rapid reviews, the study benefited from unbiased selection, reproducible and systematic synthesis of analysis. We adopted the methodology [12] used to conduct the bibliometric analysis applied to the specific context of HRM in healthcare as summarised in Fig. 1 (a customised illustration of the literature selection process). The search was based on the Web of Science database—a comprehensive and broad bibliographic database [13]. Additionally, the database is revered for its high quality and comprehensive indexing of journals in Management, Health, Biomedicine and Social Sciences, elements that are critical to our citation and influential sources analysis. To identify the key publications, knowledge gaps and to ensure transparent reporting, we relied on key elements of the PRISMA 2020 guideline. The search query captured inter-sectional elements of HRM, Healthcare and ISM. Our search was limits included journal articles, review papers and book chapters published in English between 2000–2024, yielding an initial 3139 documents and a final 477 data set for synthesis. The search, inclusion and exclusion criteria has been summarised in Table 1. The metadata of the 477 documents was exported as plaintext and imported into VOS Viewer. Based on our three research questions, the analysis parameters included RQ1- Co-citation Analysis (minimum citation threshold of 5, full-counting method, cluster parameter 1.0), RQ2- Co-occurrence Analysis of mapping conceptual structure (minimum key words occurrence of 5) and for all the three research questions, a Citation Network Analysis using Global Citation Scores (GCS). The impact indicators and impact factor metrics provide an avenue for reliably measuring science and the impact of the scientific product outlets [12]. The analysis integrated the SLR protocol with bibliometric analysis requirements to deliver a comprehensive analysis of identified literature.

**Table 1:** Paper selection criteria and results—synthesis of numbers

Review and filtering steps	Applied actions and limits	Total number of documents
Keyword search on web of science	Human Resource Management AND (“Health Workers” OR “Health Professionals” OR “Community Health Workers” AND “Public Health” OR “Primary Healthcare” OR “Public Expenditure” OR “Crisis Management” AND “Public Health” OR “Healthcare Delivery” OR “Organization and Management” OR “Healthcare” OR “Attitude of Health Personnel” OR “Health Worker” OR “Healthcare Policy”)	3139
Publication years	Exclude publications before 2000	3055
Documents type	Consider only articles, book chapters, reviews, editorial material and proceeding papers; exclude retracted papers, letters and meeting abstracts	3017
Citation topics	Select micro and meso citation topics: satisfaction, health workforce, public administration, health, healthcare policy, political science, communication	1112
Web of science categories limitations	Health policy services, management, healthcare science services, public environmental occupational health, Industrial Relations Labour, Psychology, Applied Business, Public Administration, Multi-disciplinary Sciences, Psychology Multi-disciplinary, Social Sciences Inter-disciplinary, Primary Healthcare, Social Work, Political Science, Social Issues, Social Sciences Biomedical	505
Language	Consider only documents published in English	477

*Source* WoS Data, 2024

### Study selection, inclusion, and exclusion criteria

We mapped the scientific literature in healthcare HRM based on bibliometric scientific data as per Table 1. The columns 1, 2 and 3 represent the steps taken to search, select and synthesise the literature from the database, the specific actions and limits applied, and the cumulative number of documents achieved with each step respectively.

## Results

The findings improve the understanding of the knowledge landscape based on a descriptive quantitative analysis, and synthesis of most influential studies relating to HRM, ISM and Healthcare.

### Descriptive findings

The purpose, goals and targets of both SDG 3 and SDG 8 are imperative to the future sustainability of economies. Even so, the quality, quantity, well-being and capacity of the health workforce have been debated for a long time in literature, with some raising critical managerial approaches, strategies and issues. The most cited articles were identified based on the keyword search function.

### Thematic analysis

To address RQ1(HRM factors and foundational themes that influence ISM), we performed cluster analysis based on co-citation analysis (reveals intellectual structure based on frequency of citations of papers with similar theoretical and conceptual underpinnings). The originally identified 19 clusters were generated using VOS Viewer algorithms that analysed citation strength and generated the top 6 clusters, thereafter, the authors manually conducted a qualitative analysis to synthesise the dominant topics, by iteratively reviewing titles, abstracts and for the most cited papers, the main text; [14, 15] based on the VOSViewer generated clusters, resulting to cleaned up summary as illustrated in Table 2. The identified codes were aggregated into general thematic clusters as captured under topics.

**Table 2:** Thematic clusters from co-citation analysis

Cluster	Nodes	Links	Topics	Top 3 cited papers	Period	Size %
1	9	19	HRM innovation in health, work engagement, HR capabilities, health workers behaviour	[16–18]	2013–2022	3.98
2	9	18	Lean management, empowerment, work intensification, flexibility, Multi-disciplinary HRM, workplace innovations, and care	[19, 20]	2000–2023	4.82
3	8	23	HPWP, healthcare sector, HRM practices, empowerment, work engagement, green SHRM, work attitudes, satisfaction, well-being, burn out and HCWS	[21–23]	2010–2021	4.82
4	8	32	HR management decisions, satisfaction, staffing, compassionate practices, facility design, environment, policy, research and practice	[24–26]	2007–2021	6.71
5	8	25	Leadership engagement, organisational performance, management research, HPWS, psychological empowerment, social connectedness, lean management, outcomes, social connectedness, inclusion	[27–29]	2012–2022	5.24
6	6	25	HRM, performance, transformational leadership and HR development: learning, satisfaction, management, outcomes in healthcare, controlling healthcare professionals: job attitudes, efficiency	[30–32]	2003–2020	5.24

*Minimum citations = 5 **N = 364 (100%)

Own Study, 2024 from WOS Data and VOS Viewer

### Global citation scores (GCS)

Global citation scores analysis transcend network codes, to illustrate high influence studies. Although GCS is imperative in depicting the cumulative impact of a paper, normalised GCS is a more recent and accurate measure of impact that allows for comparison of the impact of different papers, published at different times. Table 3: The top 10 most cited articles ranked by normalised GCS are related to the most significant clusters in the Citation Network Analysis thus supporting R1, RQ2 and RQ3 identifying foundational and influential studies, while providing a basis for evidence-based insights for further discussion. Table 3 illustrates the mapped papers with high GCS and their appearance in CNA. Despite being published in 2012, [27] still received the highest citation, an indicator that the issues studied on well-being and organisational performance are relevant to continuous research and improvements in the HR space.

**Table 3:** Top 10 most cited papers ranked by GCS

Rank	Title	Authors	Publication year	Journal	Appearance in CNA	GCS	Normalised GCS
1	Employee well-being and HRM organisational performance	[27]	2012	International Journal of Management Reviews	Yes	488	10.37
2	CSR and firm performance: Mediating Role of Green HRM and environmental Outcomes	[33]	2021	Journal of Business Research	No	133	6.02
3	The relationship between job stress, quality of working life and turnover intention among hospital employees	[34]	2011	Health Services Management Research	No	108	4.06
4	Increasing Employee well-being and performance: engaging leadership and HRM Practices	[28]	2021	Human Resource Management	Yes	88	3.99
5	Effective healthcare teams: defining teamwork competencies	[35]	2007	BMC Health Research Services	No	96	3.96
6	HPWS Effects on the Chinese Hospital Employees’ well-being	[36]	2013	International Journal of HRM	No	154	3.85
7	Role of HRM in reducing patient mortality in hospitals	[37]	2006	Journal of Organisational Behaviour	No	166	3.21
8	How HPWS affect the work attitudes of hospital employees and their intention to leave	[35]	2013	International Journal of HRM	No	100	2.5
9	Dutch Case Study of HPWP in the Healthcare Sector	[21]	2010	International Journal of Manpower	Yes	100	2.12
10	Patient Mortality and Employees’ Management in Acute Hospitals	[38]	2002	International Journal of HRM	No	194	1.93

*Source* Own Study, 2024 from WOS Data and VOS Viewer

## Discussion

SLR and bibliometric analysis was anchored on the perspective that HRM for health workers is not a support but a core function, a potential source of advantage, effectiveness and sustainability. The conceptualisation is based on SHRM that advances that human resource is a strategic organisational asset that drives performance. The results support the theoretical advancement and consistently show that beyond strategic asset considerations, the application of SHRM systems in health contingently depends on often overlooked aspects of internal stakeholder interest management. This section synthesises data insights from the most influential literature sources identified in our bibliometric analysis while relying on presented thematic clusters to develop an integrated perspective of HRM, ISM, SDG 3 and SDG 8.

### The knowledge structure of HRM in Healthcare

In addressing RQ1, our analysis is based on the identified six thematic clusters, also presented in Table 2. The bibliometric analysis reveals several key factors in health human resourcing that influence stakeholder management practices and performance. The top issues include turnover, compensation, well-being, satisfaction and engagement. Subject to SHRM, cluster 3 (HPWS) and cluster 6 (HRM and performance) align HR roles and practices to effectiveness. The SLR evidence that bundled HR practices that honour the management of human capital often deliver superior positive outcomes. Some of the HRM issues emerging from the influential work of [30, 32] are training practices, staffing arrangements, and performance-based compensation and reward systems.

There is a significant relationship between organisational performance and employee well-being in the healthcare sector [27]. Employee well-being evolved in SHRM, beyond traditional and administrative HRM and personnel management practices. Given the critical role of human skill, compassion and soft dynamics demanded by the nature of healthcare, our study corroborates that contingent relationship between healthcare outcomes and the well-being of workers. Consequently, we argue that better performance outcomes are realised when there are contributory high levels of employee’s well-being, and as a result, underscores the importance of HRM practices that enhance well-being.

The co-occurrence of keywords analysis (Table 4) reveals that burnout, stress, job satisfaction and turn-over are some of the most pressing HRM issues. Theoretically, this indicates the salience of well-being in SHRM. Job satisfaction is closely linked to lower turnover rates and higher performance; therefore, its strategic management is critical in responding to healthcare system challenges and facilitating the achievement of SDG 3 and 8. Job stress and quality of work-life impact turnover intentions, thus, improving job satisfaction mitigates turnover, increases retention rates and enhances performance [34]. Work engagement is another critical factor, engaged employees are more committed, perform better, and are less likely to leave their jobs.

**Table 4:** Main research topics based on co-occurrence of key words

Cluster	Key words	Total link strength	Occurrences*	Main research topics
1	Behaviour; Healthcare; Management	50; 537; 297	10; 118; 75	Management and organisational behaviour in healthcare
2	Commitment; HRM; Job satisfaction; Systems	138; 411; 212; 185	24; 79; 36; 34	Attitudes, commitment, turnover, satisfaction, quality, teamwork, motivation, performance and sustainability
3	Health policy; Strategy	39; 16; 60	8;5; 13	Policy, governance, crisis management and Strategy
4	Impact; Turn-over	410; 141;147	77; 31; 25	Communication, collaboration, technology, costs, competitive advantage, health human resources
5	Burn-out; Performance: Engagement	135; 424; 68	22; 85;11	Antecedents, challenges and demands of work engagement for health workers

*Source* Authors, 2024 from WOS Data and VOS Viewer

Based on these bibliometric patterns and SLR insights, we contend that SHRM systems for healthcare drive the outcomes of individual, organisational and SDG targets.

### Emerging managerial paradigms

In addressing RQ2, we recognise managerial dilemmas, tensions and constraints in managing the scoped HRM and stakeholder management issues in the backdrop of expectations to deliver better outcomes and sustainability. The influential sources identified elements for effective health human resource stakeholder management among the most dominant being HPWS. These systems include comprehensive HRM practices that enhance employee performance through practices like teamwork, empowerment, and continuous development. Further, [35, 36] discuss the positive effects of HPWS on employee attitudes and performance. Similarly, cluster 2 reveals that lean management and empowerment underscore efficiency. The implementation of lean management principles and employee empowerment is highlighted. This approach focuses on improving efficiency, reducing waste, and empowering frontline workers to make decisions, as [20] discussed. However, the existing co-citation by [19] fronts work intensification at the intersection of lean management. Resulting in a fundamental managerial dilemma. According to the contingency perspective, efficiency is a key organisational goal, while, employee well-being as also been identified as a SHRM asset, creating a tough balancing act for managers. Addressing burnout and managing stress are crucial for sustaining workforce performance. Organisational support and stress management practices can reduce burn-out and improve job satisfaction and commitment among healthcare workers. We contend that the managerial dynamics are constantly faced off by the demands of burn out and stress management risks, away from the typical managers’ check-list, thus reliant on cluster 5 practices of support, leadership and other operational tools.

### The intersection of SHRM and healthcare

Our study integrates HRM with the SDGs, to address RQ3. SHRM practices play a pivotal role in supporting the delivery of high-quality healthcare services. Theoretically, we advance that integrating the human capital support aspects within existing HRM practices is critical for healthcare sustainability.

Clusters 3 and 6 are anchored on High-Commitment Work Systems (HCWS) which involve practices that build a committed and competent workforce, such as extensive training, career development opportunities, and participative decision-making. We operationalise these elements in the context of SD8 (Decent work) within the framing of ensuring a stable and supportive work environment. [23] indicates that HCWS lead to better employee attitudes and lower turnover, thereby supporting sustainable healthcare delivery.

Consistent with SDG 3 (Good health and well-being), we argue that promoting equity and inclusion in the workplace is essential for delivering both personal employee well-being equitable healthcare services. Inclusive HRM practices that address the diverse needs of healthcare workers, ensuring that all employees feel valued and supported are essential [28, 29]. We position this as an imperative role of leadership in translating HR policy into action and converting resource into crucial leverage capabilities. SHRM practices, such as those that align HR policies with organisational goals, are critical for improving healthcare delivery, the cross-cutting issues being governance and service delivery approaches. SHRM can enhance patient satisfaction and the overall quality of care by ensuring that HR practices support organisational objectives [21, 24]. Innovative HRM practices, such as flexible work arrangements and continuous professional development, can help healthcare organisations respond to changing demands, maintain optimal service delivery of good healthcare to populations [15, 17].

## Conclusion

The integration of SHRM, ISM and SDGs are critical for effective and optimal healthcare delivery. As derived from the most influential revelations of bibliometric patterns and analysed sources, our study concludes that while SHRM especially HPWS are deemed to be essential, they are not independent and therefore cannot achieve success on their own. SHRM contingently relies on supportive leadership, employee well-being dynamics and a decent work environment. To harness the synergies optimally, there must be a model that integrates building a sustainable workforce that can deliver equitable and high-quality healthcare efficiently within a HCWS framework. These insights can guide HR professionals and policymakers in developing effective HRM strategies tailored to the unique challenges of the healthcare sector.

### Theoretical implications

We advance debates on the dual dynamics of sustained SHRM for healthcare by integrating both high performance and high commitment perspectives. We contribute to the ongoing conversation on SHRM as a pillar of organisational advantage while underscoring the human perspective as a crucial part of well-being, engagement and sustainability. Consistent with the contingency perspective, we spotlight leadership as a pivotal function in delivering the long-term success of both perspectives, when integrated in a single model.

### Practical implications

Our study offers evidence-based insights for healthcare managers, policy makers, and leaders.

For policy makers, who also influence health-care financing and public health workforce decisions, we build an investment case for allocating significant budgets to the well-being, training and leadership programs of health-workers. By clearly stating the intersection between SDGs and SHRM, we seek to spotlight that such allocations are strategic and not discretionary/supplementary. Health system managers can benefit by redesigning performance management tools and exercises to capture the duality of performance metrics related to HCWS and HPWS, bringing on board both efficiency and well-being and articulating the metrics to leadership as the bridge between employees and senior leadership. For healthcare professionals, we advance deeper understanding of their professional role as strategic healthcare system partners and not peripheral actors. We also promote employee’s awareness of managing burnout, stress and vouching for inclusion and participation.

## Research limitations

The study was limited to bibliometric analysis of data obtained from Web of Science; other databases could be explored. Additionally, data visualisation was done using VOS Viewer, which is widely accepted but limited to its own software parameters and algorithms. In addition, we focused on the literature published in English language between the year 2000–2024, this search strategy could potentially result in geographical, and socio-cultural bias in the interpretation and conceptualisation of the contextual health human resourcing issues.

## Data Availability

The author (s) declare that required data and material used in the manuscript development process are available. The datasets used and/or analysed during the current study are available from the corresponding author on reasonable request.

## Abbreviations

PHC: Community healthcare
SDGs: Sustainable development goals
CH: Community health
HPWS: High performance work systems
HCWS: High commitment work systems
SHRM: Strategic human resource management
